# Inactivation of the *UGPase1* gene causes genic male sterility and endosperm chalkiness in rice (*Oryza sativa* L.)

**DOI:** 10.1111/j.1365-313X.2008.03405.x

**Published:** 2008-04-01

**Authors:** Mi-Ok Woo, Tae-Ho Ham, Hyeon-So Ji, Min-Seon Choi, Wenzhu Jiang, Sang-Ho Chu, Rihua Piao, Joong-Hyoun Chin, Jung-A Kim, Bong Soo Park, Hak Soo Seo, Nam-Soo Jwa, Susan McCouch, Hee-Jong Koh

**Affiliations:** 1 Department of Plant Science and Research Institute of Agriculture and Life Sciences, Seoul National University Seoul 151-921, Korea; 2 National Institute of Agricultural Biotechnology RDA, Suwon 441-707, Korea; 3 IRRI Los Banos, Philippines; 4 Department of Molecular Biology, College of Natural Science, Sejong University Seoul 143-747, Korea; 5 Department of Plant Breeding and Genetics, Cornell University Ithaca, NY 14853-1901, USA

**Keywords:** *Oryza sativa*, genic male sterility, chalky endosperm, UGPase1, RNA*i*, complementation test

## Abstract

A rice genic male-sterility gene *ms-h* is recessive and has a pleiotropic effect on the chalky endosperm. After fine mapping, nucleotide sequencing analysis of the *ms-h* gene revealed a single nucleotide substitution at the 3′-splice junction of the 14th intron of the *UDP-glucose pyrophosphorylase 1* (*UGPase1*; EC2.7.7.9) gene, which causes the expression of two mature transcripts with abnormal sizes caused by the aberrant splicing. An *in vitro* functional assay showed that both proteins encoded by the two abnormal transcripts have no UGPase activity. The suppression of UGPase by the introduction of a UGPase1-RNA*i* construct in wild-type plants nearly eliminated seed set because of the male defect, with developmental retardation similar to the *ms-h* mutant phenotype, whereas overexpression of UGPase1 in *ms-h* mutant plants restored male fertility and the transformants produced T_1_ seeds that segregated into normal and chalky endosperms. In addition, both phenotypes were co-segregated with the *UGPase1* transgene in segregating T_1_ plants, which demonstrates that UGPase1 has functional roles in both male sterility and the development of a chalky endosperm. Our results suggest that UGPase1 plays a key role in pollen development as well as seed carbohydrate metabolism.

## Introduction

Male sterile (ms) mutants have been reported in many species of higher plants as the result of both spontaneous and induced mutations (Kaul, 1998). The mutations are associated with a range of different phenotypes, including structural aberrations such as short filaments (Mulligan *et al.*, 1994), lack of dehiscence (Dawson *et al.*, 1999) or pollen with a smooth surface (Ariizumi *et al.*, 2003), and also with functional defects associated with gametogenesis, specifically meiosis (Dawson *et al.*, 1993; Glover *et al.*, 1998; He *et al.*, 1996; Moritoh *et al.*, 2005; Peirson *et al.*, 1996; Ross *et al.*, 1997; Sanders *et al.*, 1999). All of these mutations result in non-functional pollen. A number of genes associated with male sterility have been identified in diverse plant species such as Arabidopsis (Aarts *et al.*, 1993; Ariizumi *et al.*, 2003, 2004; Thorlby *et al.*, 1997; Wilson *et al.*, 2001), wheat (Block *et al.*, 1997; Klindworth *et al.*, 2002), Chinese cabbage (Miao *et al.*, 2003), soybean (Jin *et al.*, 1998), tomato (Gorman *et al.*, 1996), sunflower (Chen *et al.*, 2006; Perez-Vich *et al.*, 2005) and chives (Engelke and Tatlioglu, 2000).

Male sterility is conditioned by either cytoplasmic-specific (CMS) or genetic (chromosomal) male sterility (GMS) genes. In rice, male sterility is classified into four major groups: male sterility caused by CMS, photoperiod-sensitive GMS (PGMS), thermo-sensitive GMS (TGMS) and other genic male sterilities (Kurata *et al.*, 2005). The CMS lines require a combination of male-sterile and fertility restorer lines to maintain a hybrid system, whereas an alteration of environmental conditions, such as day length and/or temperature, can restore fertility in PGMS and TGMS lines (Liu *et al.*, 2001; Wang *et al.*, 2003). However, the prediction and control of environmental factors, especially of temperature, is not always possible in the field. Abnormal weather can bring the temperature down below the critical level that is required to regain fertility in TGMS lines, which is simply called fertility conversion. This results in a potential problem for the seed production of two-line hybrid rice, such as the mixture of real hybrids with selfed seeds. To ensure high-quality hybrid seed production from P/TGMS lines, molecular markers can be used to help remove false hybrids from the mixture. In the past, several morphological markers, such as pale leaves (Dong *et al.*, 1995) or purple leaves (Mou *et al.*, 1995), have been employed for marking P/TGMS lines. However, removing false hybrid seedlings must be performed manually, which is labor-intensive and cannot ensure that false hybrids have been completely eliminated.

In a previous publication, Koh and Heu (1995) reported on the discovery of a new, chemically induced GMS gene, *ms-h,* and showed that it was recessive and associated with the chalky endosperm character. They suggested that the gene might be useful in a hybrid seed production system, and discussed its effectiveness compared with other systems. The *ms-h* gene was mapped to the distal region of chromosome 9 and was demonstrated to have a pleiotropic effect on the chalky endosperm (Koh *et al.*, 1999). Most reported male sterility genes are closely linked to, or have pleiotropic effects on, deleterious characteristics, making them poor candidates for use in economically viable hybrid seed production. On the contrary, because the pleiotropic effect of the recessive *ms-h* gene is expressed only in the seeds of the homozygous male-sterile (mother) plants, this character is useful for predicting which individuals will produce heterozygous F_1_ hybrid progeny, based on an examination of the seeds prior to planting.

The rice genome contains two homologous UDP-glucose pyrophosphorylase (UGPase) genes, *UGPase1* on chromosome 9 (Abe *et al.*, 2002) and *UGPase2* on chromosome 2 (GenBank accession number AF249880). The *UGPase2* gene is 80% similar at the cDNA nucleotide sequence level, and is 88% identical at the amino acid sequence level, to *UGPase1*. Both *UGPase1* and *UGPase2* are ubiquitously expressed throughout rice development, and *UGPase1* is expressed at much higher levels than *UGPase2*. Remarkably, *UGPase1* transcripts are present at higher levels in florets before flowering, suggesting that it plays a special role in rice flower development (Chen *et al.*, 2007).

In this paper, we report on the map-based isolation of the *ms-h* gene, and on the identification of a single nucleotide substitution in the *UGPase1* gene that leads to the production of nonfunctional proteins with abnormal sizes, and results in male sterility and the chalky endosperm character.

## Results

### High-resolution mapping of the ms-h gene

**Figure 1:**
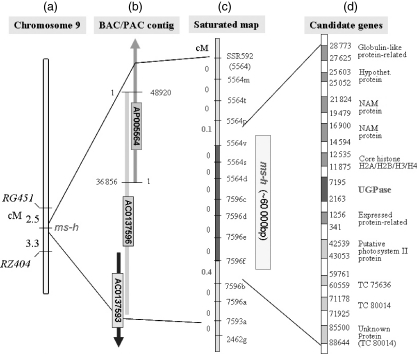
Saturated map of the region containing the ‘*ms-h*’ locus and candidate genes.
Linkage map of ‘*ms-h*’ with flanking markers on chromosome 9 (Koh *et al.*, 1999).The PAC/BAC contigs encompassing the *ms-h* gene region.The saturated linkage map of the region containing the ‘*ms-h*’ locus based on a genetic analysis of the F_2_ population (1051 plants). The *ms-h* gene was flanked by the STS (sequence-tagged site) markers, 5564p and 7596b, at a distance of 0.1 and 0.4 cM, respectively.The grey boxes indicate a total of 11 candidate genes contained in the approximately 60-kb DNA region between two STS markers, 5564p and 7596b. Sequence comparison of candidate genes between the original parent and the mutant revealed a single nucleotide substitution in the ‘*UGPase1*’ gene. NAM is the abbreviation for No Apical Meristem.

The *ms-h* gene was previously mapped to the long arm of chromosome 9 in the interval delimited by RFLP markers, RG451 and RZ404, at a distance of 2.5 and 3.3 cM, respectively (Figure 1a; Koh *et al.*, 1999). For fine mapping of the *ms-h* gene, an F_2_ population was derived from a cross between the Hwacheong *ms-h* mutant (temperate *japonica*) and Milyang 23 (an *indica*-like *Tongil*-type variety), and 1051 F_2_ plants were evaluated for phenotypic segregation of male fertility and sterility by examining spikelet fertility and chalky endosperm in F_3_ seeds. To identify additional markers closely linked to the *ms-h* gene, we designed 15 STS (sequence-tagged site) and 12 CAPS (cleaved amplified polymorphic sequence) markers based on available rice genome sequences within the interval containing the *ms-h* gene (Table 1). To identify genomic targets for CAPS marker design, we first compared publicly available rice genome sequences in the target region between the *japonica* variety, Nipponbare and the *indica* variety, 9311, using the Gramene database (http://www.gramene.org) and NCBI Blast (http://www.ncbi.nlm.nih.gov). Subsequently, only those sequences with differences in the recognition sites of restriction enzymes were used as templates for designing CAPS primers. The STS and CAPS markers were used to survey F_2_ plants, and the *ms-h* gene was found to be flanked by STS markers, 5564p and 7596b, at a distance of 0.1 and 0.4 cM, respectively. The interval spanned a region defined by two overlapping PAC/BAC clones, AP005564 and AC137596, on chromosome 9 (Figure 1b). Nine recombinant individuals were identified between markers 7596f and 7596b within an interval of 14 451 bp. Seven STS markers co-segregated with the *ms-h* locus in all the mutant plants. As a result of this map-based cloning experiment, the region containing the *ms-h* gene was narrowed down to a 60-kb region flanked by STS markers, 5564v and 7596f (Figure 1c).

**Table 1:** The PCR-based molecular markers designed for fine mapping

CAPS	Forward primer (5′→3′)	Reverse primer (5′→3′)	Fragment size amplified in *japonica* (bp)	Restriction enzyme	Originated clone
869a	CTTCCCCGAGGTAGGTGCTA	CAGGCACATCAACAATTCCA	1296	*Rsa*I	AP006548
869c	TCCAGCAGAGTCTCCATCAA	CACAGTCATCACATGCATCATT	1377	*Alu*I, *Msp*I	AP006548
RG451	TCCATAAGATCGTTCATCTGG	GTGTAAACCCTGGATGTGATG	550	*Mnl*I	AP005862
18420b	TTTTGGTCGTGACCGTGTAA	AGGCTCATATCAACGCGAAA	1311	*Alu*I, *Mnl*I, *Tru*9I,	AP006149
2505a	AAAAATCTTGGCACCAGAGG	GAATTTTGATGTGGGAGCTG	1582	*Xba*I, *Dra*I, *Sau*3AI	AP006548

STS	Forward primer (5′→3′)	Reverse primer (5′→3′)	Fragment size amplified in *japonica* (bp)	Originated clone
SSR592	ACATCATGGGCTTTCCAAAC	GCTATCCGATCGATACCTTCC	269	AP005742
5564m	CACTTTGGTTAGGCCGACTC	GCGTAAGACCTCCCTCCAAT	165	AP005564
5564t	CAGGTGACCAGGTGGAATTT	TGCCTACTTTGGGTTTGTTTG	422	AP005564
5564p	CGGATCAGCTAAGAGCGATT	ACCACGCGAGGTATGAGC	156	AP005564
5564v	TCTCCATGACCAACCTATTGC	CAAGGGAGAGTTTCCTCACG	160	AP005564
5564s	CTCTTGCCGTGCTATGTGAA	TCAAACTCCAAAACCCAAGC	396	AP005564
5564d	CCTGCCATCTCTTCAAGCAT	TCAAGTTCACACAGCCAAGT	292	AP005564
7596c	CAAAGCGGACAGAAAACGAT	TCTGGTTTTTAGCTATGCCGTA	136	AC137596
7596d	CGGCTTCTTTCCTCTTTCG	GGAGTATGAGGAGGGGAAGG	178	AC137596
7596e	TCGCTACTTTTACCGCATCC	CAAAACCAGTGGGCTACACC	221	AC137596
7596f	GAACTTTAGAAAAGGTAAGGCTTCT	CAGTTTGATTGCACCATTGC	188	AC137596
7596b	GATGACGCCCAACAATCTCT	GGACTATAGGCCGTTCCTTG	143	AC137596
7596a	TCTGAGTGGTTGGTTTGTCG	GGGTGTACTGTGGGATTTCG	178	AC137596
7593a	AAGAACATGTACCCTACGAACACA	TTTTTCTTCCTCACCAGAACAA	279	AC137593
2462d	TTCTTTTTCATGCCCCACTC	GATCCGGACAGGTTCGTTTA	1526	AC137593

### UGPase1 is the candidate for the ms-h gene

**Figure 2:**
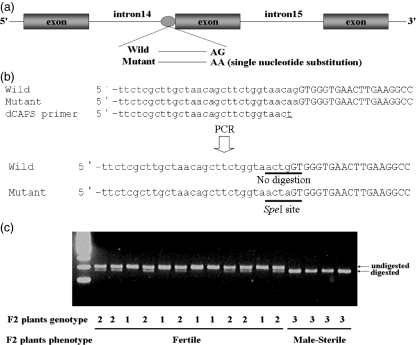
Schematic diagram of the *UGPase1* gene and derived cleaved amplified polymorphic sequence (dCAPS) marker analysis.
The mutation within the *UGPase1* gene in the *ms-h* mutant.dCAPS marker development for detection of the 1-bp substitution at the 3′-splice junction of the 14th intron. The dCAPS marker using a mismatch primer selectively generates a restriction site (*Spe*I) in the mutant, but not in the wild-type parent. Lowercase letters of sequences indicate the 14th intron and uppercase letters indicate the 15th exon.dCAPS marker genotype of F_2_ plants from Hwacheong *ms-h* mutant × Hwacheong, classified by phenotype. After digestion with restriction enzyme *Spe*I, a single, 196-bp PCR product was observed in fertile homozygotes (genotype 1), whereas in the male-sterile homozygotes (genotype 3), a shorter, 169-bp PCR product was observed, resulting from the generation of a new *Spe*I recognition site resulting from the single nucleotide substitution. In fertile heterozygotes (genotype 2), both fragments are observed. Genotype 1, *Ms-h/Ms-h*; genotype 2, *Ms-h/ms-h*; genotype 3, *ms-h/ms-h*.

Eleven candidate genes were identified in the 60-kb target interval based on *in silico* genome annotation (http://rgp.dna.affrc.go.jp; http://www.tigr.org/tdb/e2k1/osa1; Figure 1d). To identify the best candidate for the *ms-h* gene among these genes, we sequenced all 11 gene candidates in the *ms-h* mutant and in the wild-type (wt), Hwacheong, and compared them with the corresponding sequences in the publicly available genome sequence for cv. Nipponbare. This comparison identified a point mutation in the *UGPase1* gene that distinguished the *ms-h* mutant from both Hwacheong and Nipponbare. The critical polymorphism was a single nucleotide substitution of Guanine to Adenine, corresponding to the final nucleotide at the 3′-splice junction of the 14th intron of the *UGPase1* gene (Figure 2a).

To further explore the association between this single nucleotide polymorphism (SNP) in the *UGPase1* gene and the male-sterile phenotype of the *ms-h* mutant, we designed a dCAPS marker to detect the functional base substitution, and used it to trace the inheritance of the *ms-h* mutation in an F_2_ population derived from a cross between the Hwacheong *ms-h* mutant and wt Hwacheong. dCAPS analysis offers a robust and accurate tool for detecting SNPs without sequencing, and it is particularly valuable for analyzing F_2_ segregation because dCAPS markers are co-dominant and can readily distinguish heterozygotes from homozygotes (Michaels and Amasino, 1998; Neff *et al.*, 1998). We constructed a dCAPS marker consisting of a mismatch primer UGP1-CAPS-F that generated a *Spe*I site specifically in the *ms-h* mutant (Figure 2b). Using the primer set UGP1-CAPS-F and UGP1-CAPS-R a 196-bp DNA fragment could be amplified from all F_2_ plants. When the 196-bp fragments were digested with *Spe*I (recognition sequence, A/CTAGT), F_2_ plants showing spikelet sterility displayed a short fragment as a result of digestion with *Spe*I, whereas F_2_ plants showing spikelet fertility had a longer, undigested fragment (Figure 2c). Some F_2_ plants showing spikelet fertility contained both fragments, indicating that these plants were heterozygous for the alleles of both wt and mutant *UGPase1*.

To examine whether the G-to-A mutation at the 3′-splice junction of the 14th intron of the *UGPase1* was present as a natural variant in other cultivars, we performed dCAPS analysis on seven additional cultivars, including four japonicas (Ilpum, Dongjin, Nagdong and TR22183), two *Tongil* types (Dasan and Milyang 23) and one *indica* type (IR36). All seven cultivars demonstrated only one undigested fragment (data not shown). This supported our hypothesis that the 1-bp mutation identified in the *UGPase1* gene is responsible for the male sterility of the *ms-h* mutant.

### Analysis of ms-h transcripts and enzyme activity assays based on deduced amino acid sequences

**Figure 3:**
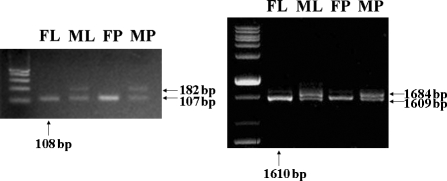
RT-PCR analysis with *UGPase1*-specific primers. As a result of each RT-PCR using the UGP1-PRT primer set (left) and the UGP1-FRT primer set (right), wild-type (wt) Hwacheong displayed only the expected size fragment in both reactions; whereas the *ms-h* mutant contained two fragments, one similar to the wt and a second, longer fragment. The same banding pattern was observed in both leaf and panicle. The arrows pointing upwards indicate the fragment size of the wt Hwacheong, and the arrows pointing left represent the fragment size of the *ms-h* mutant. Abbreviations: FL, fertile leaf; ML: male-sterile leaf; FP, fertile panicle; MP, male-sterile panicle.

As this point mutation occurred at the splice site between the 14th intron and 15th exon, we performed RT-PCR analysis of the *ms-h* mutant and wt Hwacheong using two sets of UGPase1-specific primers to investigate whether the pre-mRNA splice site was altered. One set of UGPase1-specific primers, UGP1-PRT primers, was designed from the sequences of the 14th and 15th exons, to fully span the 14th intron region, and the second set, UGP1-FRT primers, was used to amplify a full-length *UGPase1* cDNA. As shown in Figure 3, wt Hwacheong displayed only a 108-bp fragment in RT-PCR with UGP1-PRT primers, whereas the *ms-h* mutant contained two fragments, a 182-bp fragment as well as a shorter fragment that appeared identical to the 108-bp fragment observed in the wt, Hwacheong. When the shorter fragment amplified from the *ms-h* mutant was cloned and sequenced, the RT-PCR product showed a 1-bp deletion in the spliced message, compared with the corresponding sequence of the wt RT-PCR product, although this 1-bp difference was not detectable on the PAGE gel. Moreover, the 182-bp fragment amplified from the *ms-h* mutant was revealed to contain the entire, unspliced 14th intron (74 bp).

**Figure 4:**
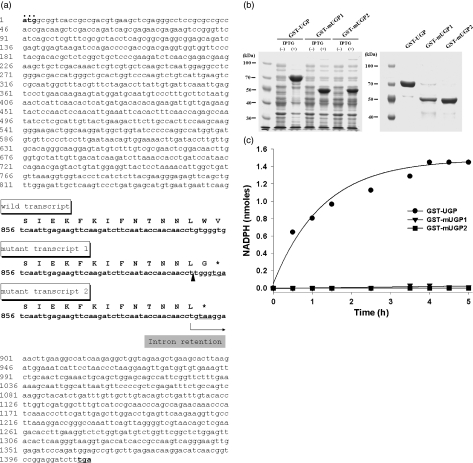
Nucleotide sequence of the *UGPase1* open reading frame, and alignment of the nucleotide and deduced amino acid sequence of the 14th exon 3′-region derived from wild-type (wt) Hwacheong and from the *ms-h* mutant. Nucleotide numbering starts from the start of translation; the protein sequence is derived from the nucleotide sequence. Wild transcript denotes the *UGPase1* transcript of wt Hwacheong, whereas mutant transcripts 1 and 2 represent the 1-bp deleted transcript and the 74-bp inserted transcript found in the *ms-h* mutant, respectively. The deduced amino acid sequences of the two *ms-h* mutant transcripts with abnormal sizes demonstrate that both the 1-bp deletion and the 74-bp insertion cause a frame shift and generate stop codons, resulting in truncated 299- and 298-aa proteins, instead of the full-length 469-aa protein. The start codon is set in **bold** with a row of dots above it. The underlined sequences and asterisk indicate termination codons. The 1-bp deletion site is marked by an arrowhead.SDS-PAGE of recombinant proteins induced with isopropyl-beta-D-thiogalactopyranoside (left) and purified-recombinant UGPase1 proteins (right). The molecular weight of glutathione S-transferase (GST) (23-kDa)-tagged recombinant UGPase1 proteins agrees with the estimated values of 74 and 56 kDa for the full-length UGPase1 protein (469 aa) and the two C-terminal deleted proteins (299 and 298 aa).Activity assays of three recombinant UGPase1 proteins. The formation of NADPH was calculated from the absorption changes at 340 nm monitored for 5 h using an NADPH molar extinction coefficient of 6.22 × 10^3^ m^−1^ cm^−1^. •, GST-UGP, GST-tagged recombinant protein containing full-length UGPase1; ▴, GST-mUGP1, GST-tagged recombinant protein containing C-terminal deleted 299-aa UGPase1; ▪, GST-mUGP2, GST-tagged recombinant protein containing C-terminal deleted 298-aa UGPase1.

The deduced amino acid sequences of the *ms-h* mutant transcripts displaying two abnormal sizes suggests that both the 1-bp deletion and the 74-bp insertion cause frame shifts that generate two independent stop codons in the process of translation, resulting in truncated 299- and 298-aa proteins, instead of the 469-aa protein encoded by the wt *UGPase1* transcript (Figure 4a). To further confirm whether two C-terminal deleted UGPase1 proteins of the *ms-h* mutant are nonfunctional, we performed enzyme activity assays *in vitro*. The *UGPase1* cDNAs encoding full-length protein of the wt Hwacheong and two truncated proteins of the *ms-h* mutant were amplified by PCR with each specific primer, and were transformed into *Escherichia coli* after vector construction. Glutathione S-transferase (GST)-tagged three-recombinant proteins were purified and separated by SDS-PAGE (Figure 4b). UGPase activity assays were performed with recombinant proteins by monitoring the NADPH formation at 340 nm. As expected, enzyme activity of GST-UGP containing the full-length UGPase1 protein appeared, whereas both GST-mUGP1 and GST-mUGP2 did not show any enzyme activity, indicating that two C-terminal deleted proteins were nonfunctional (Figure 4c). Thus, this single base substitution in the splice site found in the *ms-h* mutant appears to cause unstable splicing, leading to the presence of two mature transcripts, both with abnormal sizes and, because of the corresponding stop codons, the mRNA transcripts are translated into a truncated and a nonfunctional UGPase1 protein, respectively.

### RNAi-mediated silencing of the UGPase1 gene causes male sterility

**Figure 5:**
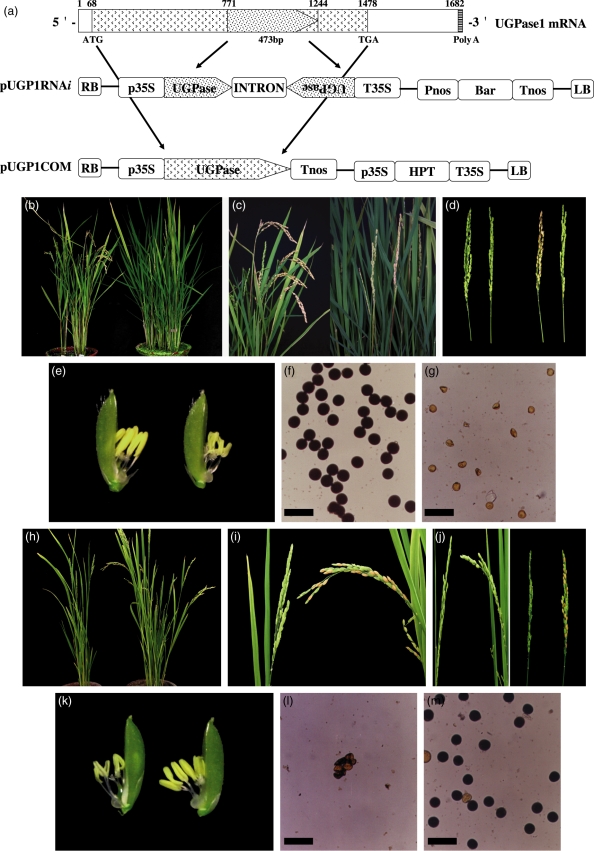
Transgene constructs and phenotypes of transgenic plants.
Schematic diagrams of the pUGP1RNA*i* construct for double-stranded RNA interference and the pUGP1COM construct used for the complementation test. In pUGP1RNA*i*, the 473-bp gene-specific fragment of the *UGPase1* gene was linked with the intron in both antisense and sense orientations, such that the transcripts were expected to create a dsRNA stem with a single-stranded loop. Phenotype of UGPase1-RNA*i* plants (b–g).Hwacheong plants after ripening: containing the empty vector (left) and transformed by pUGP1RNA*i* (right).Photograph (c) is the double enlargement of a part of the photo (b).Panicles of a vector-transformed plant and a UGPase1-RNA*i* plant at anthesis (left) and after ripening (right).Flower and anther morphology of a vector-transformed plant (left) and UGPase1-RNA*i* plant (right) at the heading stage. To view the anthers, the lemma was ripped off.I_2_-KI staining of pollen grains from a vector-transformed plant at the heading stage, showing the presence of normal, round and starch-filled grains.I_2_-KI staining of pollen grains from a UGPase1-RNA*i* plant at the heading stage, showing the presence of abnormal, small and non-stained grains caused by the lack of starch. Phenotypic complementation by introduction of the *UGPase1* gene (h–m).Phenotype of Hwacheong *ms-h* mutants after ripening: plants containing the empty vector (left) and complemented by the introduction of pUGP1COM (right).Photograph (i) is the triple enlargement of a part of the photo (h).Panicles of a vector-transformed plant and the complemented plant at anthesis (left) and after ripening (right).Flower and anther morphology of an empty vector-transformed plant (left) and the complemented plant (right) at heading stage.I_2_-KI staining of pollen grains from a empty vector-transformed plant at heading, showing the presence of abnormal and non-stained grains.I_2_-KI staining of pollen grains from the complemented plant at heading. This photograph shows the presence of normal grains, indicating the restoration of fertility. The scale bar corresponds to 100 μm.

To confirm that the *UGPase1* gene is causally related to male fertility, we generated UGPase1-RNA*i* transgenic plants by exploiting double-stranded RNA (dsRNA)-mediated interference to silence the target gene (Baulcombe, 2002; Moritoh *et al.*, 2005; Prasad and Vijayraghavan, 2003). The RNA*i* construct included 473-bp of the gene-specific sequence, corresponding to the full-length *UGPase1* cDNA, which was linked with the intronic sequence in the antisense and sense configurations, and then placed under the control of the constitutive 35S promoter (Figure 5a). The UGPase1-RNA*i* construct was introduced into calli derived from wt Hwacheong immature embryos by *Agrobacterium*-mediated transformation, with an empty vector used as a control. Thirty-three independent transformants were regenerated, and the presence of the RNA*i* construct was confirmed by PCR using Bar-F and Bar-R primers (data not shown). At the spikelet ripening stage, five of the transformed lines displayed low fertility, and two lines were male sterile (Figure 5b–e). Moreover, these transformed lines showed pleiotropic developmental abnormalities similar to the Hwacheong *ms-h* mutant phenotype, including reduced culm length and retarded growth (Table 2).

**Table 2:** Morphological characteristics of transgenic plants^a^

Line	Heading date	Culm length (cm)	Panicle length (cm)	Spikelet fertility
wt Hwacheong	August 23	87.4	18.3	Fertile
r23^b^	August 22	63.8	18.1	Sterile
Difference	ns	**	ns	
Hwacheong gms	August 21	62.5	18.0	Sterile
c10^c^	August 22	85.5	18.2	Fertile
Difference	ns	**	ns	

a The original T_0_ plant was grown in the field by crown division.

b r23: *UGPase1* silenced transformant.

c c10: *UGPase1* complemented transformant.

** Significant at the 0.01 probability level.

ns, not significant.

**Figure 6:**
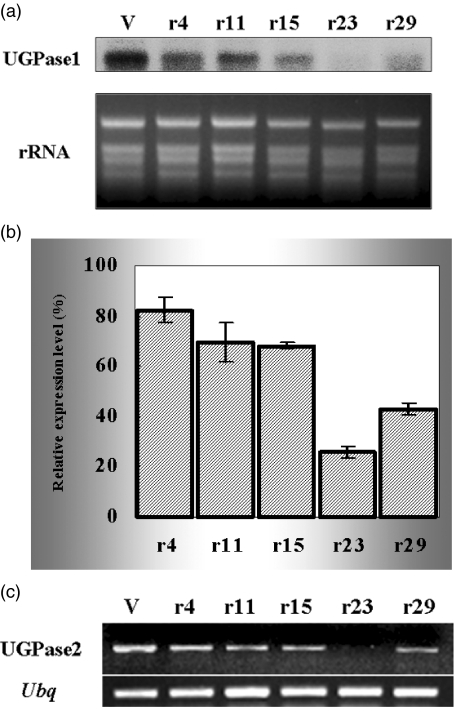
Expression level analysis of *UGPase1* and *UGPase2* genes in UGPase1-RNA*i* plants.
Northern blot analysis of *UGPase1* gene expression. The upper panel shows the RNA gel blot probed with the *Not*I fragments of UGP1 *i* pGEMT containing the 473-bp gene-specific region of the *UGPase1* gene. The lower panel shows ethidium bromide stained rRNA as a loading control.Quantitative RT-PCR analysis of *UGPase1* gene expression. The expression value was normalized with a *ubiquitin* control, and the results represent the average values of duplicate experiments shown as relative expression levels compared with empty vector-transformed plants.Semi-quantitative RT-PCR analysis of the expression of the *UGPase2* gene. Amplification of the *ubiquitin* gene was used as a control. V, empty vector-transformed plant; r4, r11, r15, r23 and r29: UGPase1-RNA*i* plants.

When pollen viability was compared in high- and low-fertility lines, by staining for starch with I_2_-KI solution, the five low-fertility transformants showed light pollen staining as compared with empty vector-transformed plants that displayed normal starch accumulation, and pollens from the two male-sterile transformants (r23 and r29) did not stain for starch (Figure 5f,g). If the low or no-staining phenotypes of UGPase1-RNA*i* transformants were caused by the introduced dsRNA, we would expect to see reduced *UGPase1* transcription levels in these transgenic plants. When Northern blot analysis was used to examine *UGPase1* expression levels in RNA samples harvested from spikelets at the booting stage in transgenic lines, it could be seen that transcription was most severely suppressed in the two male-sterile transgenic lines (r23 and r29), and was partially suppressed in the low-fertility transformants, compared with empty vector-transformed plants (Figure 6a). Subsequently, we examined the expression of *UGPase1* transcripts in more detail in seven transgenic lines using real-time quantitative RT-PCR analysis. First-strand cDNAs that were reverse-transcribed with oligo (dT) were used as the template for the quantitative PCR analysis. Results were computed to show relative expression levels in UGPase1-RNA*i* transformants compared with a vector-transformed plant using *ubiquitin* as a standard. As can be seen in Figure 6b, *UGPase1* transcriptional levels were slightly suppressed in the low-fertility lines (r4, r11 and r15), whereas the levels were severely reduced in the ms transformants, to 26% in r23 and 43% in r29. To determine whether the expression of a dsRNA interference construct towards *UGPase1* affects the expression of *UGPase2*, we analyzed *UGPase2* expression by semi-quantitative RT-PCR of the total RNA extracted from spikelets. As shown in Figure 6c, *UGPase2* transcription in most RNA*i* transformants was just slightly suppressed, but *UGPase2* transcripts of the r23 line were completely suppressed, similar to the suppression pattern of *UGPase1* transcripts.

Taken together, the results indicate that the RNA interference of *UGPase1* causes male sterility in proportion to the transcriptional suppression of *UGPase1*, and that the endogenous UGPase mRNAs, including *UGPase1* and *UGPase2*, are degraded globally in UGPase1-RNA*i* transformants, leading to developmental growth retardation. The existence of homologous UGPase genes in rice raises the question as to whether each has a unique and independent function, or whether they share related or redundant functions. The incomplete co-suppression of *UGPase1* and *UGPase2* in RNA*i*-silenced transformants in this study implies that there may be a complementary interaction between the two homologous genes, despite the fact that virtually nothing is known about an interaction between the two homologous *UGPase* genes in rice.

### Transgenic complementation of ms-h mutation

To further confirm that the point mutation in the *UGPase1* gene causes male sterility in rice, we complemented the *ms-h* phenotype by introducing an overexpression construct containing the wt *UGPase1* sequence into homozygous *ms-h* mutants (Figure 5a). An empty vector was again introduced as a control. Transformants containing the complementation vector were selected on hygromycin, and 29 transgenic lines were regenerated. PCR screening using HPT-F and HPT-R primers identified 11 transgenic lines that contained the expression construct, and these were grown in a greenhouse and investigated for spikelet fertility and other morphological characteristics at maturity.

**Figure 7:**
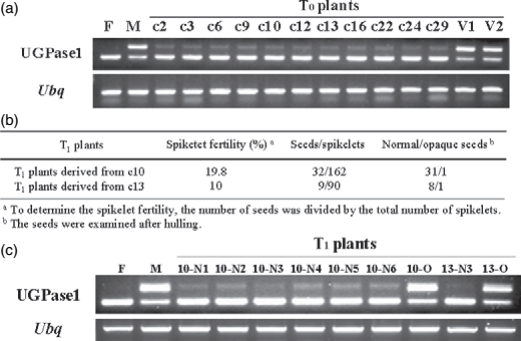
Molecular analysis of the *UGPase1* gene complemented plants (T_0_) and T_1_ plants.
RT-PCR analysis of T_0_ plants.Spikelet fertility and the ratio of normal to opaque seed of T_1_ plants derived from T_0_ plants (c10 and c13).RT-PCR analysis of T_1_ plants derived from T_0_ plants (c10 and c13). The upper panel shows a gel electrophoresis pattern resulting from RT-PCR using the UGP1-PRT primer set. The lower panel shows gel electrophoresis following RT-PCR of the *ubiquitin* gene as a control. F, wild-type plant (fertile Hwacheong); M, Hwacheong *gms* mutant; c2, c3, c6, c9, c10, c12, c13, c16, c22, c24 and c29, complemented plants; V1 and V2, empty vector transformed plants; 10-N1, 10-N2, 10-N3, 10-N4, 10-N5 and 10-N6, T_1_ plants derived from normal seeds of the c10 transformant; 10-O, T_1_ plant derived from opaque seeds of the c10 transformant; 13-N3, T_1_ plant derived from a normal seed of the c13 transformant; 13-O, T_1_ plant derived from an opaque seed of the c13 transformant; *Ubq*, ubiquitin.

Figure 5h–m shows that the introduction of the wt *UGPase1* gene complements the mutant phenotype. This finding is confirmed by the formation of filled grains, pollens that stain clearly with I_2_-KI solution and the normal formation of anthers and fertile panicles. The number of filled and empty spikelets was counted on two representative panicles per plant. Overexpression of the *UGPase1* gene in transgenic *ms-h* plants resulted in spikelet fertility that ranged from 33.4% to 10.2%. Although the degree of fertility restoration differs among plants, the occurrence of filled seeds in the *ms-h* background is a significant indicator of complementation. Moreover, all of these transformants recovered a wt Hwacheong phenotype with normal morphology (Table 2). RT-PCR analysis with the UGP1-PRT-F and UGP1-PRT-R primer set showed amplification of a single, strong-intensity fragment that was similar in size to that seen in the fertile wt Hwacheong control (Figure 7a). When this fragment was subcloned and sequenced, it was found to include a mixture of two fragments: one that was identical to the wt *UGPase1* gene transcript and one that corresponded to the abnormal fragment derived from the *ms-h* mutant. In the meantime, complementation of *UGPase1* has no effect on the expression of *UGPase2* (data not shown). Accordingly, this complementation test further confirmed that functional disruption of the *UGPase1* gene is responsible for male sterility in rice.

### The ms-h gene has a pleiotropic effect on chalky endosperm

Koh *et al.* (1999) previously reported on the co-segregation of the *ms-h* gene and the development of a chalky endosperm. To confirm that the chalky endosperm results from a pleiotropic effect of the *ms-h* gene, we evaluated both male-sterile and male-fertile transgenic progeny to determine whether opaque seeds were always associated with *ms-h*. In the case of UGPase1-RNA*i* transformants, two male-sterile transgenic T_0_ lines (r23 and r29) were crossed with wt Hwacheong as the male parent to obtain F_1_ progeny. Seven F_1_ seeds were planted and observed for spikelet fertility and the occurrence of opaque seeds (chalky endosperm) after ripening of the spikelets. From a cross between r23 × Hwacheong, only one F_1_ progeny was obtained, which was male sterile. This result was predicted based on the Northern blot analysis of the r23 line that demonstrated the efficiency with which the RNA*i* construct suppressed *UGPase1*. At the same time, two out of six F_1_ progenies derived from r29 × Hwacheong were male sterile, one had a low spikelet fertility (21.5%), and three were male fertile, with spikelet fertilities ranging from 78.1% to 80.8%. Remarkably, three fertile or partially fertile F_1_ progenies produced a small number of opaque F_2_ seeds, at an average ratio of one-fiftieth (Table 3). This suggests that expression of functional *UGPase1* may be intermittently suppressed in these second-generation RNA*i* transgenic lines, or that the RNA*i* construct is imperfectly transmitted from one generation to the next.

**Table 3:** Spikelet fertility and the ratio of normal to opaque seeds of UGPase-RNA*i* transformant F_1_ progenies

No.	Cross combination	Spikelet fertility (%)^a^	Seeds/ spikelets	Normal/opaque seeds^b^
r23	r23 × Hwacheong	0	0/259	0
r29	r29 × Hwacheong	0	0/191	0
r29	r29 × Hwacheong	0	0/130	0
r29	r29 × Hwacheong	21.5	83/386	81/2
r29	r29 × Hwacheong	80.8	156/193	153/3
r29	r29 × Hwacheong	78.1	171/219	168/3
r29	r29 × Hwacheong	78.5	128/163	128/0

a To determine the spikelet fertility, the number of seeds was divided by the total number of spikelets.

b The seeds were examined after hulling.

The T_1_ seeds harvested from 11 T_0_ transgenic lines produced in the complementation test were also examined for chalkiness after hulling. Chalky grains were segregated in two (c10 and c13) of the 11 plants (Figure 7b). The opaque T_1_ seeds harvested from c10 and c13 transformants were planted with other normal T_1_ seeds to verify the pleiotropism with male sterility. After maturing, we confirmed the co-segregation of the *ms-h* gene and seed opaqueness, based on phenotypic examination and molecular analysis of UGPase1-RNA expression patterns (Figure 7c). These results clarify that the *ms-h* gene has a pleiotropic effect on the chalky endosperm, which is consistent with the previous study (Koh *et al.*, 1999).

## Discussion

### A single nucleotide substitution within a splice site generates abnormal-size transcripts as a result of unstable splicing

In this study, we showed that the male-sterile phenotype observed in the *ms-h* mutant resulted from a point mutation within the *UGPase1* gene. Splicing depends on the presence of signal sequences in the pre-mRNA. In almost all genes the first two nucleotides at the 5′-end of an intron are GT, and the last two at the 3′-end are AG (Green, 1991; Moore and Sharp, 1993). According to this GT-AG rule for RNA splicing (Cai *et al.*, 1998; Isshiki *et al.*, 1998), it is reasonable to expect that a single base change from G to A, where the first base of the 15th exon is a G, would cause a one-base downstream shift of the AG site at the 3′-end of the 14th intron in the *UGPase1* gene. The result of this SNP is the formation of a 1-bp deleted transcript resulting from alternative splicing (Figure 4a). However, the formation of a 74-bp inserted transcript indicates that pre-mRNA splicing of the *UGPase1* gene in the *ms-h* mutant is an unstable process. Although introns are ubiquitous and share a high degree of structural/sequence similarity across species, the signals that specifically define splice sites are not completely understood. Two models for splice site selection have been suggested in the initial recognition of exon/intron borders: exon definition and intron definition. Although most of the early studies of splice site mutants in vertebrates favored the exon definition of splicing (Berget, 1995), initial reports from plants tend to favor the intron definition (Goodall and Filipowicz, 1989; Lou *et al.*, 1993; McCullough *et al.*, 1993). The escape from splicing according to the intron definition may be a step towards the interpretation of the 74-bp intron insertion in the *UGPase1* transcripts reported in the *ms-h* mutant in this study.

### The point mutation of the UGPase1 gene causes the loss of UGPase activity

UGPase presents in all prokaryotic and eukaryotic organisms. It catalyzes the reversible conversion of Glc-1-P and UTP into UDP-Glc (UDPG) and pyrophosphate (PP*i*), a key precursor for polysaccharide synthesis (Feingold and Avigad, 1980;Kleczkowski, 1994). The putative three-dimensional structure of the barley UGPase monomer is bowl-like, with an active site positioned in a central groove (Kleczkowski *et al.*, 2004). This shape is common for AGPase and UGPase, and perhaps for all pyrophosphorylase-like proteins (Peneff *et al.*, 2001). The active site of UGPase contains several amino acid residues that have been shown to be important for substrate binding and catalysis of the enzyme. For example, five lysyl residues (Lys263, Lys329, Lys367, Lys409 and Lys410) participate in substrate binding and catalysis in potato (Katsube *et al.*, 1991; Kazuta *et al.*, 1991). Other results also showed that several lysine residues are necessary for UGPase activity, although positions are somewhat variable among species (Eimert *et al.*, 1996; Pua *et al.*, 2000). Interestingly, rice also has similar positioning of five lysine residues (Lys257, Lys263, Lys323, Lys329, Lys361 and Lys367) as in potato, except for additional Lys440 for substrate binding in rice. In addition, position 168 (NQS) and 307 (NLS) are putative glycosylation sites, and position 420 (SER) is a phosphorylation site (Sowokinos *et al.*, 2004). In addition, previous results suggested that oligomerization of UGPase plays a regulatory role in any process requiring UDPG as a substrate, and that the C-terminus is responsible for oligomeric conformation (Geisler *et al.*, 2004). Incidentally, the mutant *UGPase1* encodes two C-terminal deleted 299- and 298-aa proteins, instead of one functional full-length (469-aa) protein. Thereby, we assume that the truncated two proteins do not have enzymatic activity. In fact, our enzymatic assay showed that these two mutant proteins have no UGPase activity (Figure 4c), supporting the hypothesis that the C-terminus is required for the enzymatic activity of UGPase.

### UGPase plays a key role in pollen and endosperm development

Most male-sterile mutants are controlled by monogenic recessive genes, and have defects in sporogenic tissues, tapetal cells, pollen mother cells, microspores and/or pollen at the pre-meiotic, meiotic and post-meiotic stages of anther and pollen development (Singh, 2003; Twell, 2002). Many male-sterile lines are characterized by a perturbed carbohydrate metabolism (Dorion *et al.*, 1996). Carbohydrates are considered to play a critical role in anther and pollen development. They are not only energy sources that sustain growth but they also take part in cell-wall biosynthesis during pollen development (Clément and Audran, 1995; Goetz *et al.*, 2001). UDPG, a key substrate/product of the enzyme for carbohydrate metabolism in both the source and sink tissues, is used directly or indirectly in the biosynthesis of cell-wall polysaccharides, reflecting the key role of UDPG as a precursor for cell-wall biogenesis (Gibeaut, 2000). An unloading pathway via the functional coupling of cell-wall invertase with a monosaccharide transporter is prominent in symplastically isolated pollen cells (Ji *et al.*, 2005; Oliver *et al.*, 2005; Roitsch *et al.*, 2003), and the subsequent conversion of Glc-1-P metabolized from apoplastically cleaved sucrose into UDPG by UGPase is a vital process for pollen cell-wall biosynthesis. These results indicated that UGPase participates in an essential process for pollen development. More recently, it has been reported that rice UGPase is essential for pollen callose deposition, and its co-suppression results in a TGMS (Chen *et al.*, 2007). Our previous results showed that pollen development in the *ms-h* mutant was arrested at the binucleate or trinucleate microspore stage because of uneven meiosis, and implied interference with cell-wall formation during pollen meiosis (Koh and Heu, 1995). Thus, our findings support that UGPase is a key component that controls pollen development.

Our previous study also showed that starch granules in the endosperm of *ms-h* mutants are more roundish, polyhedral and smaller than those of wt Hwacheong. The starch structure of the *ms-h* mutant has a higher frequency of long glucose chain amylose and a shorter branching of amylopectin than wt Hwacheong (Sohn *et al.*, 1997). Starch is synthesized by apoplastic or symplastic pathways (Kleczkowski *et al.*, 2004), and the ADP-Glc (ADPG) that is required for its synthesis is provided via two mechanisms (Denyer *et al.*, 1996). First, it is synthesized via the cytosolic ADP glucose pyrophosphorylase (AGPase), in which case a transporter is required to transfer ADPG into the plastid. Second, it is synthesized via the plastidial AGPase, in which case a supply of plastidial Glc-1-P is required (Denyer *et al.*, 1996). However, starch biosynthesis in the endosperm cells of cereals such as maize and barley mostly starts with the cytosolic synthesis of ADPG followed by the subsequent import of this compound into the storage plastid, which is dependent on an extra plastidial AGPase (Denyer *et al.*, 1996). Besides, the production of UDPG by UGPase is coupled to the activity of cytosolic AGPase in the cytosol of cereal seed endosperm, which implies that UGPase directly regulates ADPG levels by affecting its synthesis by AGPase (Kleczkowski, 1994). *Chlamydomonas* mutants with lesions in the pathway of ADPG synthesis, which presumably have reduced levels of ADPG, lack the long-chain fraction of amylopectin that is present in normal starch (Van den Koornhuyse *et al.*, 1996).

Therefore, our study on the endosperm of *ms-h* mutants with short branching of amylopectin provides clues indicating that the opaque phenotype arises from the alteration of the starch structure by the insufficient supply of long amylopectin chains, as a result of the ADPG synthesis reduction caused by the disorder of UGPase. The interaction between UGPase activity and starch in the opaque phenotype must be closely related, although the mechanism remains unclear. Therefore, the way in which UGPase participates in carbohydrate metabolism during endosperm development is worthy of further study.

## Experimental procedures

### Plant materials and genotype evaluation

A male-sterile mutant, Hwacheong *ms-h*, was induced via chemical mutagenesis using *N*-methyl-*N*-nitrosourea from a Korean *japonica* cultivar, Hwacheongbyeo (Koh and Heu, 1995). The F_2_ population used for fine mapping was derived from a cross between the Hwacheong *ms-h* mutant (*japonica*) and Milyang 23 (*tongil*-type rice, derived from an *indica* × *japonica* cross, and which was similar to *indica*). F_2_ plants (1051) were classified as either male sterile or fertile (wt) based on an examination of spikelet fertility, and to distinguish heterozygotes from homozygous wt plants, F_3_ seeds harvested from fertile F_2_ plants were evaluated for the presence of a chalky endosperm after hulling.

### Genetic mapping

Total genomic DNA was extracted from the leaves of both parents and from each F_2_ individual using the method of McCouch *et al.* (1988). Based on results from a prior mapping experiment, closely linked RFLP (restriction fragment length polymorphism) markers were used to identify DNA sequences within the *ms-h* region using an *in silico* approach (http://www.gramene.org; http://rgp.dna.affrc.go.jp; http://www.tigr.org/tdb/e2k1/osa1; http://www.genome.arizona.edu). To fine-map the *ms-h* gene, 15 STS and 12 CAPS markers were developed based on available rice genome sequence data. The STS and CAPS primers used in this work, along with the corresponding restriction enzymes for the CAPS markers, are listed in Table 1. PCR products were digested completely with specific restriction enzymes, and were then size-separated on 1–2% agarose gels containing 0.15 μg ml^−1^ ethidium bromide and 0.5× Tris-Borate-EDTA running buffer.

Linkage analyses were performed with the segregation data in the F_2_ populations using map maker version 3.0 (Lander *et al.*, 1987). Genetic distances between markers were calculated in Kosambi centi Morgans (cM).

### Sequence alignments and dCAPS analysis

Overlapping DNA fragments across the *ms-h* region were amplified by PCR. PCR fragments were then purified and analyzed by direct sequencing with a Big Dye Terminator Cycle sequencing kit using an ABI 377 sequencer (Applied Biosystems, http://www.appliedbiosystems.com). The results of sequencing were aligned with the original parent. For dCAPS analysis, PCR amplification with the primer set UGP1-CAPS-F (5′-TTCTCGCTTGCTAACAGCTTCTGGTAACT-3′) and UGP1-CAPS-R (5′-ATCAACTTCCTGTGAATACCAACTGCTTT-3′) was performed using 10 ng of extracted DNA in a total volume of 25 μl containing 1X reaction buffer, 0.5 mm deoxyribonucleotide triphosphate, 0.4 μm of each primer, and 1 U of *Taq* DNA polymerase (Bioneer, http://www.bioneer.com). A total reaction of 35 cycles was programmed for 30 sec at 94°C, 30 sec at 65°C and 1 min at 72°C in a Thermal Cycler (Bio-Rad, http://www.bio-rad.com). Each PCR product (5 μl) was digested with *Spe*I in a total volume of 20 μl at 37°C overnight. After digestion, 5 μl of each digest was electrophoresed in a 3% agarose gel.

### RT-PCR and amino acid annotation

Total RNA was isolated using the SV Total RNA Isolation kit (Promega, http://www.promega.com) following the manufacturer's instructions. A 1-μg aliquot of total RNA was reverse-transcribed using an oligo (dT) primer and an M-MLV Reverse Transcriptase kit (Promega). Of the synthesized first-strand cDNAs, 2% was used for PCR analysis with two sets of *UGPase1*-specific primers: for target region amplification, UGP1-PRT-F (5′-CCCTGATGAGCATGTGAATG-3′) and UGP1-PRT-R (5′-TCAGCTTCTACCAGCCTCTTG-3′) primers were used; for full-length cDNA amplification, UGP1-FRT-F (5′-CATATCTCCCGTCCTTTC-3′) and UGP1-FRT-R (5′-ATGAAATACAACGCCCTTGG-3′) primers were used. The amplification reaction was carried out using the following conditions: 5 min at 94°C, 35 cycles of 1 min at 94°C, 1 min at 60°C and 2 min at 72°C, with a final extension step of 10 min at 72°C. Amplification products were recovered and sequenced. The amino acid sequences of RT-PCR products were deduced and compared with the original parents using NCBI BLAST (http://www.ncbi.nlm.nih.gov/blast/Blast.cgi).

### Preparation of recombinant UGPase proteins and enzyme activity assay

The *UGPase1* cDNAs encoding the full-length protein of the wt Hwacheong and the two truncated proteins of the *ms-h* mutant were amplified by PCR and inserted into pGEX4T-1 (Amersham, http://www.amersham.com) to produce the GST-UGP containing full-length UGPase1 (469 aa), the GST-mUGP1 containing the C-terminal deleted 299-aa protein and the GST-mUGP2 containing the C-terminal deleted 298-aa protein. Three constructs were transformed into *Escherichia coli* BL21/DE3 (pLysS) cells. The transformed cells were treated with isopropyl-beta-D-thiogalactopyranoside to induce recombinant protein expression. The recombinant proteins were purified according to the supplier's instructions. The protein concentrations were determined by the Bradford assay (Bio-Rad).

UGPase activity assays were performed with the recombinant proteins of GST-UGP, GST-mUGP1 and GST-mUGP2 using a one-step spectrophotometric method (Sowokinos *et al.*, 1993). Reaction mixtures (pH 7.5) contained (in 1 ml): 5 μmol of MgCl_2_, 0.6 μmol of NADP, 1 μmol of UDP-glucose, 1 U of phosphoglucomutase, 1 U of glucose-6-phosphate dehydrogenase, 20 μmol of Cys, 80 μmol of glycylglycine, 0.02 μmol of Glc-1,6-diP and 1 μg (0.1 μg μl^−1^) of a recombinant protein. Reactions were initiated with 2.5 μmol of PP*i*. The formation of NADPH (340 nm) was monitored continuously at 30°C until the reaction rate was no longer linear. All assays were run with minus PP*i* blanks to correct for any contaminating NADPH production.

### Vector constructs and rice transformation

To generate the UGPase1-RNA*i* construct for *UGPase1* gene suppression, a 473-bp fragment of *UGPase1* cDNA was amplified using first primers UGP1-RNA*i*-F (5′-AAAAAGCAGGCTACCACCTGATCCATAACCAG-3′) and UGP1-RNA*i*-R (5′-AGAAAGCTGGGTGTTTGATGGGTTTGTTCTGG-3′), and was subcloned into pGEM-T (Promega). This construct was denoted as UGP1 *i* pGEM-T and its sequence was verified. The UGP1 *i* pGEM-T was amplified using second primers attB1 (5′-GGGGACAAGTTTGTACAAAAAAGCAGGCT-3′) and attB2 (5′-GGGGACCACTTTGTACAAGAAAGCTGGGT-3′), and the resulting attB-PCR products were cloned into the Gateway^TM^ pDONR 201 cloning vector, which carries two recombination sites (attL1 and attL2), by BP clonase reaction (Invitrogen, http://www.invitrogen.com). Subsequently, these entry clones with *UGPase1* were inserted in opposite directions into two regions, each flanked by recombination sites (attR1 and attR2) in the destination vector, pB7GWIWG2(II) (VIB-Ghent University, Belgium), using an LR clonase reaction (Invitrogen; http://www.invitrogen.com). The resulting RNA*i* construct was denoted as pUGP1RNA*i*. For the complementation test using the *UGPase1* gene, a PCR-amplified *UGPase1* full-length cDNA was digested with *Sac*I and inserted into the pCamLA overexpression vector, a pCambia 1300-modified vector containing a 35S promoter and Tnos terminator. The resulting overexpression construct was denoted pUGP1COM. *Agrobacterium* strain LBA 4404 harboring pUGP1RNA*i* and pUGP1COM was used to transform rice calli induced from the mature embryos of the normal Hwacheong and Hwacheong *ms-h* mutants, respectively, according to the method described by Hiei *et al.* (1994). UGPase1-RNA*i* plants were regenerated from transformed calli by selecting for phosphinotricin resistance, and the transformants for the complementation test were selected for hygromycin resistance. The regenerated plants were confirmed by PCR analysis with each antibiotic resistance-specific primer: for the *Bar* gene, Bar-F (5′-CATCGCAAGACCGGCAACAGGATTCAA-3′) and Bar-R (5′-GCTCCACTGACGTTCCATAAATTCCCC-3′) primers were used; for the *HPT* gene, HPT-F (5′-GTAAATAGCTGCGCCGATGG-3′) and HPT-R (5′-TACTTCTACACAGCCATCGG-3′) primers were used.

### Pollen and spikelet fertility

Pollen fertility was determined at anthesis using a 1% iodine-potassium iodide (I_2_-KI) solution, as described by Shinjyo (1969). The numbers of dark blue (stainable) and reddish brown (unstainable) pollen grains in each individual were counted under an optical microscope. Plants with <5% stainable pollen and zero seed setting of bagged panicles were classified as sterile, and all others were regarded as fertile. At the same time, fertility/sterility was confirmed by self-pollination tests.

### Real-time quantitative RT-PCR and Northern blot analysis

For real-time quantitative RT-PCR analysis of RNA*i* plants, QuantiTect^TM^ SYBR Green PCR kit (Qiagen, http://www.qiagen.com) and the Rotor-Gene 2000 (Corbett Research, http://www.corbettlifescience.com) were used according to the manufacturer's instructions. RNA isolation from spikelets at the booting stage was carried out using the TRI-ZOL^TM^ reagent from Invitrogen. A 1-μg aliquot of total RNA treated with DNaseI (Invitrogen) was reverse-transcribed using an oligo (dT) primer and AMV Reverse Transcriptase (Promega). Of the synthesized first-strand cDNAs, 10% were used for PCR analysis with different sets of gene-specific primers: for *UGPase1*, real-RNA*i*-UGP1-F (5′-CCCTGATGAGCATGTGAATG-3′) and real-RNA*i*-UGP1-R (5′-CTGCAGTTTCGAGTTGCAGA-3′) primers were used; for *ubiquitin*, RUB2-F1 (5′-AATCAGCCAGTTTGGTGGAGCTG-3′) and RUB2-R1 (5′-ATGCAAATGAGCAAATTGAGCACA-3′) primers were used as a control (Wang *et al.*, 2000). For semiquantitative RT-PCR of *UGPase2* cDNA, primer pair UGP2-F (5′-TCATCAGATCAGCGTGAAGC-3′) and UGP2-R (5′-GCCCACTCACAAGGAGAAAA-3′), based on the 5′- and 3′-untranslated regions of rice *UGPase2*, were used (Chen *et al.*, 2007). For Northern blot analysis, a 10-μg aliquot of total RNAs was separated by electrophoresis in a 1.5% (w/v) formaldehyde agarose gel and then transferred to a Hybond-N^+^ nylon membrane (Amersham). The membrane was hybridized with ^32^P-radiolabeled partial *UGPase1* cDNA probes, a *Not*I fragment of UGP1 *i* pGEMT and was then washed using standard procedures (Sambrook and Russell, 2001).
